# The Advanced Prototype of the Geohydroacoustic Ice Buoy

**DOI:** 10.3390/s20247213

**Published:** 2020-12-16

**Authors:** Leonid Sobisevich, Vadim Agafonov, Dmitriy Presnov, Valentin Gravirov, Dmitry Likhodeev, Ruslan Zhostkov

**Affiliations:** 1 Schmidt Institute of Physics of the Earth, Russian Academy of Sciences, 123242 Moscow, Russia; sobis@ifz.ru (L.S.); presnov@physics.msu.ru (D.P.); vvg@ifz.ru (V.G.); rus@ifz.ru (R.Z.); 2 Department of Physical and Quantum Electronics, Moscow Institute of Physics and Technology, 141700 Moscow, Russia; agvadim@yandex.ru

**Keywords:** molecular–electronic seismometer, geohydroacoustic ice buoy, data logger, ice-class drifting antenna, geophysical monitoring in arctic seas

## Abstract

The new-generation geohydroacoustic buoy prototype is designed for simultaneous acquisition of acoustic, hydroacoustic, and seismoacoustic data in various environmental conditions, including onshore and offshore boreholes, yet is specifically targeted for operation in Arctic seas as an element of the distributed ice-class drifting antennas. Modular structure of the geohydroacoustic ice buoy incorporates the advanced data logger and a combination of sensors: vector–scalar hydroacoustic (0.01–2.5 kHz) accelerometer, broadband molecular–electronic (0.03–50 Hz) velocimeter, as well as optional hydrophones. The distinguishing feature of the geohydroacoustic buoy is its low power consumption responsible for consistent autonomous operation of the entire measurement system for at least one week. Results of continuous laboratory tests carried out at the geophysical observatory of the Geophysical Survey of the Russian Academy of Sciences (GS RAS) in Obninsk are presented. It has been confirmed via comparative analysis of recorded time series featuring microseismic noise and teleseismic earthquakes that the prototype well meets the high standards of modern seismology.

## 1. Introduction

The recently approved Strategy for the Development of the Arctic Zone of the Russian Federation defines an overall set of measures aimed at solving the main problems of the development of the Arctic zone and ensuring the interests of national security with a planning horizon up to the year of 2035, taking into account the peculiarities of climatic and geo-ecological conditions. The integrated approach to the development of natural resources in the Arctic, on the one hand, and the preservation of the environment in a changing climate, developed in the framework of this study, is essentially linked with the improvement of existing and development of new geophysical technologies for exploration and monitoring of hydro-carbon reserves in the ice-covered Arctic seas. To solve these problems in the Arctic, the Russian earthquake monitoring system is being created [1] for seismic and infrasonic monitoring of glaciers. This will make it possible to follow the activity of outlet glaciers, which will ensure increased navigation safety in northern seas [2,3,4].

Recent seismoacoustic studies [5,6], including those carried out within the framework of various international projects in the Arctic, suggest an increasing interest in this region, the expansion of the range of tasks to be solved and the involvement of methods that provide operational information in the process of fundamental study of the Arctic waters so to obtain the local parameters of the water layer, ice cover, surface layer of the atmosphere and heterogeneous structures of the seabed. It is natural to assume that the further development of systems of complex geological–geophysical and hydroacoustic observations will ultimately lead to their unification within the framework of information-measuring networks for multi-parameter operational analysis and forecasting of the changing situation in the Arctic.

One of the most promising areas for the development of work in the interests of creating systems for integrated seismic hydroacoustic monitoring is the improvement of passive, eco-friendly seismic methods in relation to the conditions of an ice-covered shallow sea, which are implemented through the use of distributed ice-based antenna systems. Recent achievements in this field [7] have made it possible to simultaneously study the characteristics of the seafloor, water layer, and ice cover by using seismic and combined vector–scalar hydroacoustic receivers to measure the total geohydroacoustic field [8].

The main element of the distributed antenna system is a seismohydroacoustic ice-based buoy, which has a modular structure and can be equipped with various sensors, including hydrophones, vector hydroacoustic receivers, triaxial seismometers, etc. The standard design of the buoy implies the presence on board of a multichannel digital datalogger featuring the satellite time and location services according to available global positioning systems, wireless interfaces for communication with other buoys of the ice-based antenna system and autonomous power supplies capable of operating in Arctic conditions. A working model of a seismohydroacoustic ice-based buoy was developed by scientists and engineers from the Schmidt Institute of Physics of the Earth of the Russian Academy of Sciences and the JSC “Concern Sea Underwater Weapon—Gidropribor” along with the corresponding patent (Sukhoparov et al., 2016). Within the scope of this study, five experimental model devices were created, functionally ready for testing of their operational indicators. The main focus was on developing and applying an autonomous information-measuring system with a novel sensing element that would be embedded in ice and operate as a seismometer/velocimeter.

Readers may have a legitimate question: how is the presented buoy better than existing similar systems? What advantages does it have? Of course, today many types and modifications of seismometers have been developed, including passive, “classic” active ones with feedback, built on the basis of MEMS microchips [9] or molecular electronic technics. According to the principle of reading signals, the sensor can be molecular–electronic, electromechanical, or working with a liquid inertial mass [10]. They all have their own advantages and disadvantages.

In our system, we decided to use seismic sensors with liquid inertial mass, or in other words, molecular–electronic, as sensitive elements. This type of sensor gives the developed system a number of significant advantages. The use of liquid as inertial mass eliminates the design of devices from moving parts. It provide all system with increased reliability in operation, do not require special transportation conditions, are characterized by low energy consumption, operate in a wide temperature range, are practically insensitive to installation angles and do not require fixing or centering the mass. Buoys can be successfully installed both on and under the ground, on the seabed or frozen into the ice. Of course, the first systems of molecular–electronic seismometers with electrodynamic feedback were developed long before the advent of our buoy. The first industrial molecular electronic seismometer was created in 2002 and was successfully tested at the Albuquerque Seismological Laboratory (ASL) in the same year. Its serial production began in 2003 [11], as a result of which hundreds of seismometers of the EP300-SP400-EP105 [12] family were manufactured, which are successfully operated in many parts of the world to this day. Based on the experience of using such sensors and the proprietary developments of the R-sensors company, molecular–electronic sensors with force–balance feedback were created for our buoys [13]. Its presence made it possible to improve stability, expand the dynamic and temperature range, reduce nonlinear distortions and guarantee a flat transfer function in the operating frequency range of the device. We should be especially noted that the developed buoys represent the first development of geophysical sensors capable of successfully operating in the extreme conditions of the Far North. They are not afraid of being frozen into the ice; they have a sufficiently strong hull that even allows them to be lowered under water to a depth of about 300 m. The buoys are all-in-one devices, capable of functioning autonomously for a long time, as well as carrying out "fast measurements", which is especially important when mobile teams of scientists work in the Arctic. For example, when exploring the polar islands, when the time spent by the research groups is strictly limited in time and every minute of research is very important.

## 2. Operational Principles and Installation of the Measurement Module

**Figure 1:**
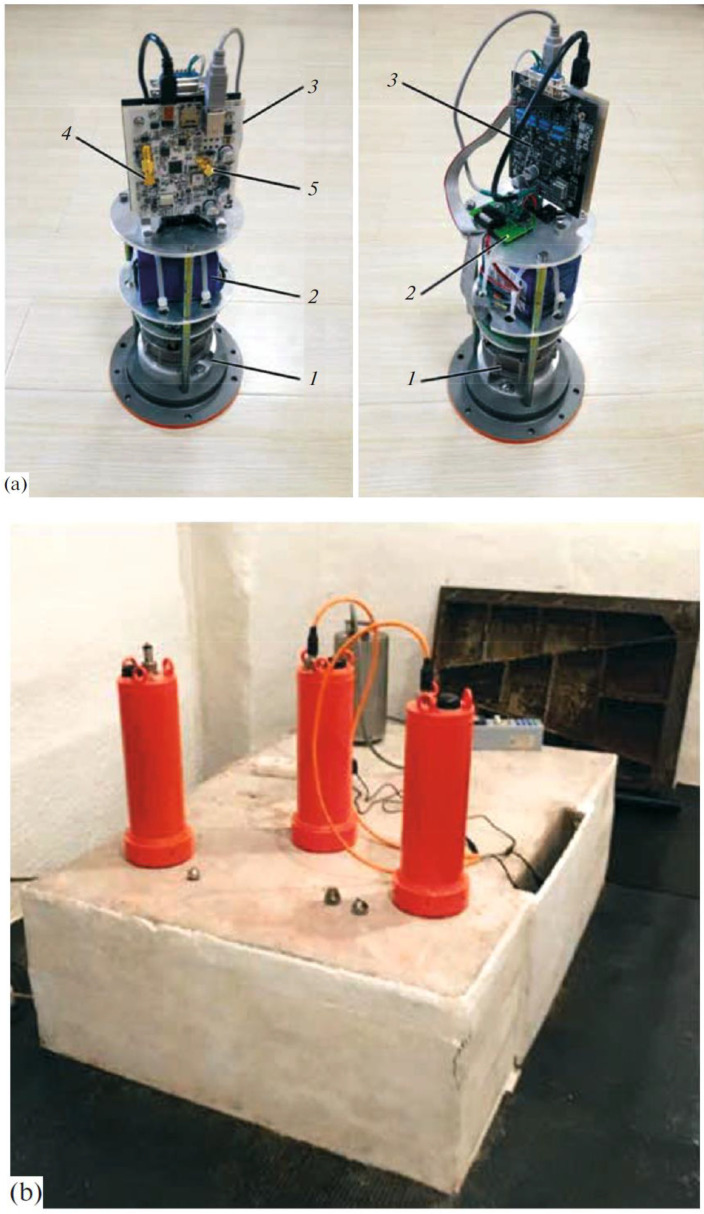
Design of geohydroacoustic module (**a**) and external view of buoy placed on pedestal in Obninsk, 30 August 2017 (**b**). (1) Analog molecular–electronic vertical seismic sensor; (2) lithium ion battery with interface board; (3) 24-bit seismic data acquisition system; (4) USB/WiFi antenna connector; (5) external GPS antenna connector.

The geohydroacoustic measuring buoy is designed for autonomous recording of seismic, hydroacoustic, and, as needed, acoustic signals during several weeks; data accumulation and transmission via a wire or wireless interface; remote checking of proper operation; and combination of several modules into a unified seismic network. The device functionally consists of three main modules (Figure 1a): a CME-4211V analog single-component molecular–electronic measuring instrument (1), a power supply unit ensuring operation of all system nodes (2), and an NDAS-8224 seismic data recorder (3). The design is patent protected (Agafonov et al., 2018). Table 1 shows the main characteristics of the developed buoys. By default, the buoy is equipped with a one-component analog seismic sensor, which is capable of registering only the vertical component of seismic movements. Further, in the article we will consider and test only this type of buoys.

Analog seismic sensor (1) operates on the principles of molecular–electronic transfer [13,14]. Molecular electronic sensors, like their recent predecessors (chemotronic sensors) use an electrolyte as a sensitive inertial mass. Modern molecular electronic seismometers based on them have a linear acceleration output signal in a wide frequency and dynamic range [15]. In the developed buoys, a highly concentrated aqueous solution of potassium iodide KI (with the lower limit of the temperature range of −15 ℃) or lithium iodide LiI (with the lower limit of the temperature range −55 °C) and a small amount of molecular iodine I_2_ is used as the electrolyte. The movement of the electrolyte along the channel creates an electric current that can be measured in some way and compared with the movement of the device base. In the sensors we used, the conversion of the electrolyte movement into an electrical signal occurs on the internal system of platinum electrodes installed inside the electrolyte container. Four installed electrodes, separated by microporous dielectric separators, form an electrochemical cell. A small constant voltage is applied between the cell electrodes. Thus, the passage of current through the cell will be largely determined by the hydrodynamic motion of the electrolyte caused by external mechanical disturbances. It should be borne in mind that the rate of chemical reaction on the electrodes is usually much higher than the rate of delivery of reacting substances to them. As a result, a concentration gradient of these substances appears in the area of the electrodes. Charge transfer in a stationary electrolyte is carried out by molecular diffusion from one electrode to another. If the electrolyte is set in motion under the influence of inertial forces, then along with molecular diffusion, convective transfer of ions occurs, which sharply changes the rate of delivery of reacting substances to the electrodes and, accordingly, the current passing through the electrode system changes. In this case, the diffusion and convective transfer of the active component determine the current flowing in the system. Thus, the movement of the electrolyte creates proportional variations in current. A distinctive feature of this type of sensors is a high conversion coefficient, which is expressed in their significant electrical response to any mechanical stress, which significantly exceeds the noise of any used electronic components (even at low levels of input influences). As a result, it is possible to provide a high signal-to-noise ratio for the entire measuring path.

Electrode-potential-based seismic sensors are extremely reliable: they do not require any special tools, methods for transport and installation, maintenance, arrestment, or centering of mass; they are very suitable for field measurements and rapid rollout of a seismic network [13]. Table 2 lists the sensor parameters.

The power source is based on lithium ion elements. The overall size of the battery packed into the single housing is 51 mm × 56 mm × 77 mm, and the total battery capacity at room temperature is around 7000 mAh. The battery’s nominal voltage is 9–12.6 V. Since digital block (3) and analog molecular-electronic measuring device (1) have different voltages (+5 and +12 V, respectively), a special interface board is provided, which converts the battery voltage to +5 V for powering the digital recorder.

Recorder (3) is designed for use in battery-powered autonomous data acquisition systems. It is based on a 24 bits sigma-delta ADC with 22 effective bits. The sample rate can be chosen from the following range values: 1, 10, 50, 100, 250, 500, and 1000 Hz. The receiver module has a small power consumption (less than 300 mW during recording operations), which makes it possible to substantially increase its autonomous operation time for the given device dimensions.

For data transmission and system configuration, USB cable or WiFi connection (connector (4) in Figure 1a) is used; the 32 GB internal memory allows long-term data recording in the autonomous mode. The recorder has chains for feeding test signals to the seismic sensors, as well as a power of ±2.5 V. The integrated GPS module makes it possible to synchronize seismic events with accurate universal time (UTC), automatically start and stop recording on a set schedule, and identify the spatial location point of the measuring module on the Earth’s surface for individualization of modules in the seismic network.

The geohydroacoustic measuring module is mounted in a durable dust-proof housing with a protection rating no lower than IP67. The upper lid sports the module’s external combined WiFi/GPS and a hermetically sealed multifunction PC-10 port. This port makes it possible to charge the module’s battery, read data from the recorder, and program the digital module; there is also a LED indicator of the device’s operation and the presence of a GPS satellite signal fixed by the module. When the LED indicator shield is connected to the port, power is supplied from the internal battery.

The developed software performs the following functions: device list display, status parameters of devices (coordinates, clock synchronization, orientation, etc.), seismogram display for one or several devices, display and saving of seismograms to the device’s memory.

## 3. Laboratory Bench Testing of Hydroacoustic Measuring Module

### 3.1. Earthquake Recording

The main indicators of the broadband sensors in the ice-class buoys were studied observationally in underground boxes on measuring stands of the GS RAS geophysical complex (Obninsk). A multi-hour observation experiment (from August 30 through September 8, 2017) made it possible to compare the amplitude and phase frequency characteristics of the buoy with precision seismometers. The precision seismometer was a Streckeisen STS-1V/VBB velocimeter—the main instrument of the continuously operating Obninsk seismic station (OBN) which is a part of Global Seismograph Network—IRIS/IDA; in addition, the data also were analyzed with a Güralp CMG-3ESP velocimeter.

**Figure 2:**
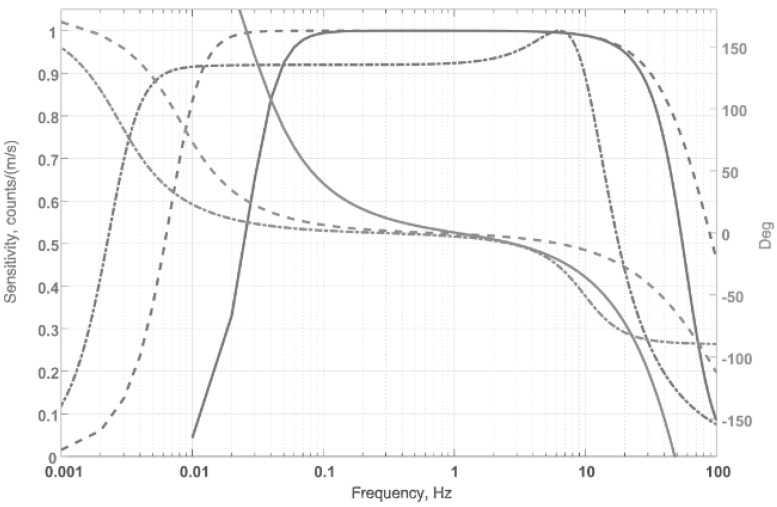
Typical amplitude-frequency response (AFR) and phase-frequency response (PFR) of Streckeisen STS-1V/VBB (dash-dotted line), Güralp CMG-3ESP (dotted lines), and CME-4211V (solid lines) devices.

Figure 2 compares the frequency characteristics claimed by the manufacturers with the frequency characteristics of the CME-equipped geohydroacoustic buoy. The Streckeisen and Güralp devices have an acceptable sensitivity up to periods of 250 and 100 s, respectively; the available data are limited from above by the Nyquist rate, 10 and 20 Hz, respectively. This study considers the frequency range of 0.03–10 Hz. The seismic stations are located in an area shielded from extraneous noise by a strip of forest. The geophysical stand (Figure 1b) was at a depth of ∼20 m, the temperature was around 12 ℃, and high humidity was noted.

**Figure 3:**
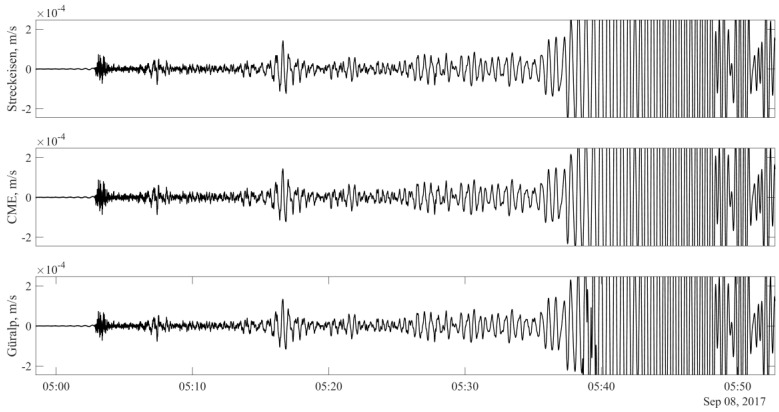
Comparison of records obtained by Streckeisen, Güralp, and CME devices in frequency range of 0.03–10 Hz for 8.1 magnitude earthquake in Mexico at 04:49 UT on 8 September 2017.

**Figure 4:**
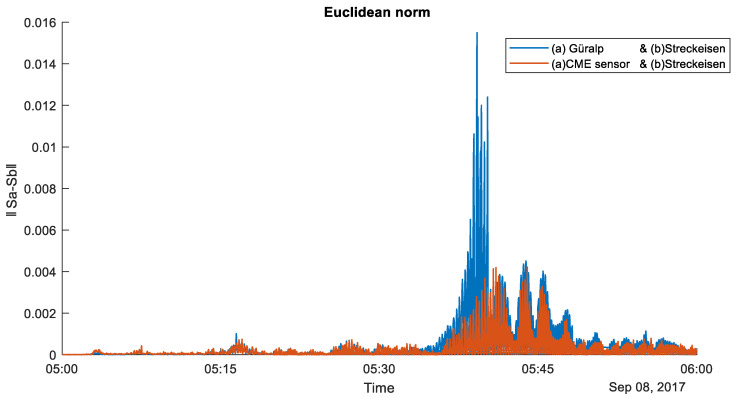
Euclidean norm that represent similarity between two pairs of signals recorded: Streckeisen and Güralp (blue), Streckeisen and CME sensor (red) in frequency range of 0.03–10 Hz for the same data shown on Figure 3. (Earthquake in Mexico at 04:49 UT on 8 September 2017).

The most informative event that occurred during the measurement period was the 8.1 magnitude earthquake in Mexico at 04:49 UT on 8 September 2017. Figure 3 shows the synchronous (in terms of maximum values) record of the event from all three receivers, filtered in the frequency range of 0.03–10 Hz, taking into account the frequency characteristic of the CME device shown in Figure 2. The records of the other devices were not calibrated, since their amplitude-frequency (AFR) and phase-frequency (PFR) responses can be considered linear in the indicated frequency band. The lower (compared to the benchmark devices) sensitivity of the CME at periods greater than 20 s is noteworthy. Removing CME sensor response by applying corresponding transfer function solves the problem, making the records perfectly identical as it shown on Figure 3. To quantify the degree of similarity between two signals, the Euclidean norm of a one-hour records difference where calculated (see Figure 4). As a result, the following values were obtained $\|S_{a}-S_{b}\|=0.008$ and $\|S_{a}-S_{c}\|=0.014$, where S with indexes *a, b,* and *c* correspond to signals showed on Figure 3 from top to down. Very small value for CME sensor confirms that geohydroacoustic ice buoy not inferior to a high-class observatory instrument Streckeisen STS-1V/VBB in strong earthquakes recording tasks. In addition, necessary note that Güralp velocimeter seismogram was off the scale for high signal values due to the reorder settings, which leads to signal distortions and high value of calculated norm. This allows us to make a well-founded conclusion that the developed geohydroacoustic buoy meets the high standards of modern seismometric equipment and is not inferior in its parameters to the best competing equipment samples.

### 3.2. Influence of Changes in Ambient Temperature

The influence of changes in ambient temperature on the characteristics of seismic instruments has long been known [16,17]. With the advent of electronic versions of such equipment, the problem of temperature influence has not disappeared anywhere, since any useful electrical signals always coexist with electrical noise. Electrical noise is a fluctuation of current and voltage and can be caused by a wide variety of phenomena. When anyone considering current and voltage fluctuations, thermal noise is usually separated (Nyquist noise or, in other words, Johnson noise) [18,19], that is, fluctuations that occur in any thermodynamically equilibrium system, and shot noise [20], which occurs when charge carriers accidentally cross some potential barrier. The mechanism behind this noise is well understood and the level of fluctuations is predictable. In most systems, they determine the minimum amount of fluctuation that can be obtained. Unfortunately, real electronic circuits often generate more noise than expected for thermal and shot noise [21]. Despite the variety of mechanisms of occurrence, statistical properties, and conditions for the manifestation of excess fluctuations, there is no single classification of such noises. Usually, certain types of noise are distinguished, for example, generation-recombination noise [22], current distribution noise [23], explosive noise [24], flicker noise [25], and others.

There is an opinion [24,26] that the main reason for the appearance of increased electrical noise of seismic devices is the instability of the parameters of electronic components and their damage. As a proof, there is information that before assembling STS seismometers [27], an additional multistage check of their electronics components is always carried out. Further, in the designs of many seismic devices, components with a tolerance no more than 1% are used.

It is especially difficult to take into account the effect of changes in ambient temperature on the characteristics of precise devices. In this case, due to the fact that inside the volume of any device there are heat-generating elements (dissipative circuits, active, and passive electronic components), and also due to the fact that the seismometer’s elements have different thermal inertia coefficients, temperature gradients are always observed [16,17]. The resulting interference can arise because, firstly, the linear dimensions of structural elements (even those having the same temperature coefficient of linear expansion) vary unevenly with temperature, which can cause their movement and bending. Secondly, convective air flows appear which can have a direct effect on the sensitive elements of instruments, for example, on a seismometer’s pendulum [24]. Accounting and analysis of such noises is a big problem. In addition to the coefficient of thermal inertia and the temperature coefficient of linear expansion, it is necessary to take into account the thermal conductivity and heat capacity of the elements, their dimensions and geometry, the shape and size of the air cavities inside the device, the location and power dissipation of the electronic components, and so on. In general, noise caused by temperature gradients cannot be taken into account. It should be noted here that simple thermostating of the entire device helps in most cases to protect only from external temperature drops. However, it turns out to be completely useless in the presence of internal local sources of heating.

The transfer function of almost any seismic instrument can also change under the influence of fluctuations in ambient temperature [28]. Moreover, the aging of electronic components and the associated change in their parameters from nominal values have a significant effect on the form of the transfer function. Even the scatter of parameters within the tolerance band can lead to significant differences in the transfer functions of the devices. In this regard, it becomes necessary to determine the characteristics of each device instance and to correct the obtained data [29], including requirements regular calibration of seismic equipment. 

The influence of changes in environmental parameters on the noise characteristics of the basic seismic instruments is described in [17], where a detailed analysis of both noise sources and possible ways of dealing with them is given.

**Figure 5:**
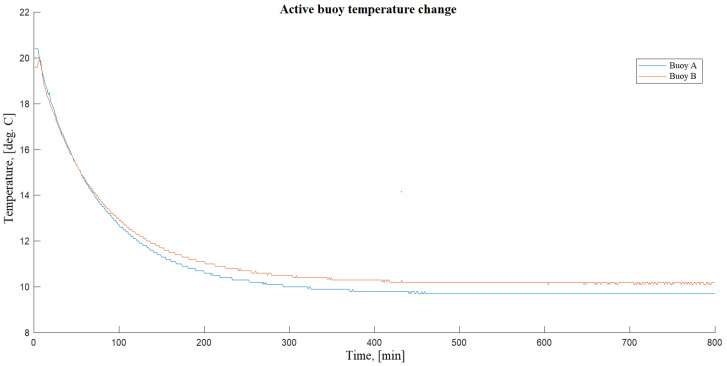
The process of cooling of the internal temperature of the buoys.

As we noted before, geohydroacoustic buoys are constructed on the use of a low-noise molecular–electronic sensor (see Table 2) with the sensing filled electrolyte. However, the use of a liquid as a working fluid can limit its use. It is obvious that these devices have fundamental limitations to their functioning at low temperatures. In the first models of buoys, the electrolyte freezes at a temperature of about −15 ℃. It should be mentioned that today we can use new frost-resistant variations of sensitive elements with the lower limit of the operating temperature range of the electrolyte up to −40 ℃. During the experiment (described in Section 3.1) temperature, measurements were also carried out to assess the effect of temperature on the transfer characteristic of the geohydroacoustic buoys. The measurements were carried out with a 100 Hz sample frequency. As was noted earlier, geohydroacoustic buoys have ability to monitor and record the temperature of their internal space. The internal temperature was sampled once a minute. Since the air temperature on the surface in the area of this seismic station on that day was about +20 ℃ and the buoys were switched on for registration immediately after their descent and installation in the adit, the records obtained during the cooling of the buoys from 20 to 10 ℃ (see Figure 5) that can be used to investigate possible changes in their transfer characteristics.

**Figure 6:**
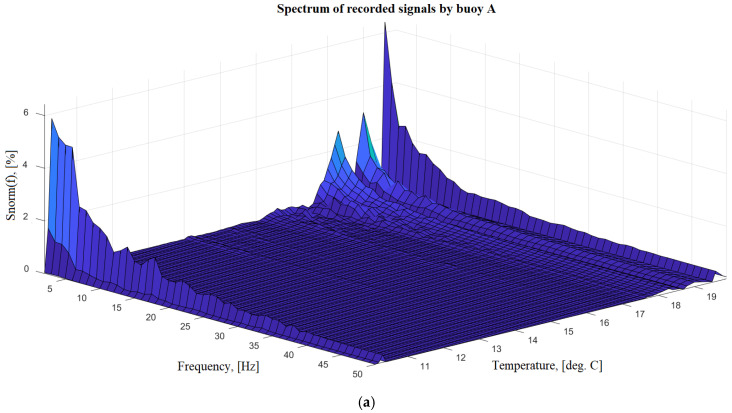

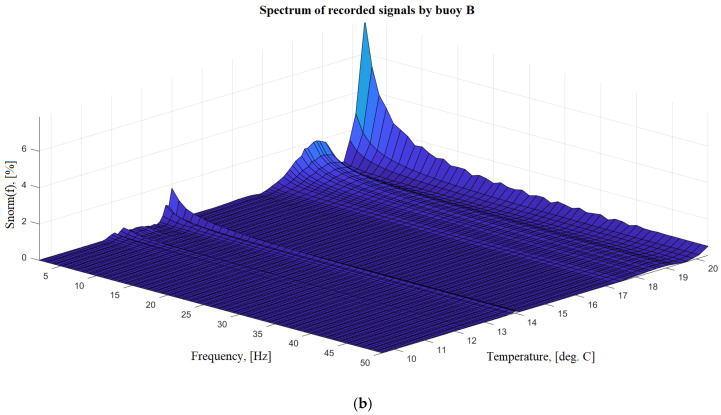
Changes in the spectral composition of the recorded signals by buoys A (**a**) and B (**b**) depending on the internal temperature.

To obtain control records of seismic noise parameters, in parallel with the installed two geohydroacoustic buoys (named in experiment as “A” and “B”), the records made by Guralp CMG-3ESP installed at the same place were used. The analysis of the data recorded by the Guralp CMG-3ESP made it possible to conclude that the seismic noise in the area of the seismic station during the experiment was stationary. In addition, because during the cooling of the geohydroacoustic buoys were recorded no seismic events, we will assume that the average level and frequency composition of the recorded seismic noise was constant. Figure 6a,b show the results of spectral analysis of signals recorded from buoys A and B, respectively, depending on the observed internal temperature. These figures show the spectra normalized to the general frequency background, so you can easily see the results that somehow stand out in the frequency and temperature ranges. Considering the figures, it can be noted that both investigated geohydroacoustic buoys have an insignificant change in the frequency composition of the recorded signals and, accordingly, we can talk about some change in their characteristics depending on the ambient temperature. Both investigated buoys have a slight increase in the transfer characteristic in the low-frequency part, which begins to manifest itself at a device temperature of more than +17 ℃.

The conducted research shows the presence of an insignificant effect of the ambient temperature on the amplitude-frequency characteristics of these geohydroacoustic buoys, which confirms the previously stated need for constant monitoring of the external and internal temperatures of such devices. For a more accurate and detailed study of this dependence, it is planned to investigate a larger number of buoy samples in the future, as well as significantly expand the investigated temperature range.

### 3.3. Buoy Field Tests

**Figure 7:**
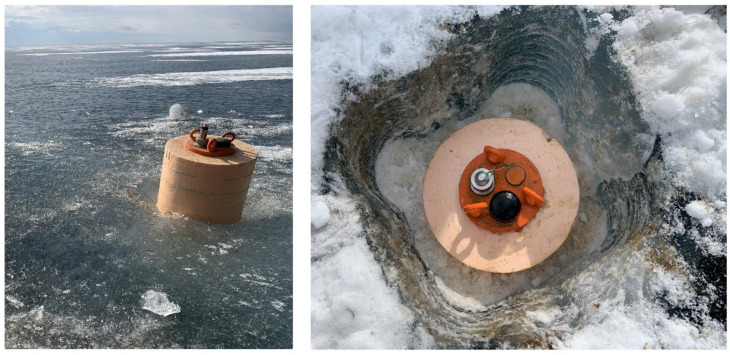
Installation of geohydroacoustic ice buoys during a field experiment on the ice of Ladoga Lake.

**Figure 8:**
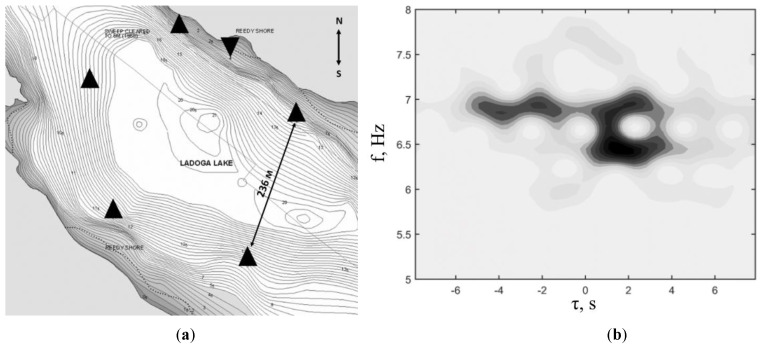
(**a**): Diagram of unique tomographic measurements performed using geohydroacoustic buoys (indicated by triangles, where the inverted triangle indicates the central base measuring station) from the ice cover taken in 2018; (**b**): spectrogram of the cross-correlation function of seismoacoustic noise, averaged over 8 h, recorded by buoys frozen in ice.

During 2018, a tomographic experiment was carried out in ice conditions, during which the operability and reliability of the developed geohydroacoustic buoys were confirmed in practice (see Figure 7). A diagram of the location of the experiment and an example of the experimental data obtained is shown in Figure 8. Based on the continuous data recorded in the offline mode, bottom surface waves of the Scholte (Stoneley) type, hydroacoustic modes and flexural-gravity modes of the ice cover were identified and studied, which made it possible to remotely monitor parameters of all three media formed in the "lithosphere–hydrosphere–ice cover–atmosphere" system. The obtained scientific results made it possible to formulate and patent the method of ice-based technology for hydrocarbon exploration in the Arctic.

**Figure 9:**
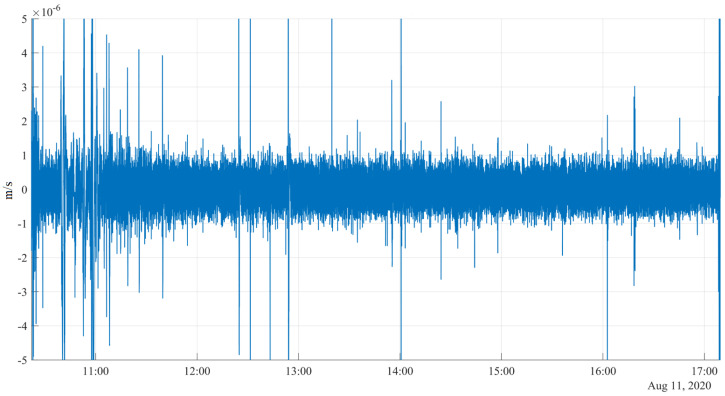
The seismogram recorded on near the crater edge on the Avachinsky volcano.

In 2020, in Kamchatka region, an experiment was carried out to install several buoys on the summit of the Avachinsky volcano in the immediate vicinity of the crater. Unique records of the somma of the crater of the Avachinsky volcano were obtained (see Figure 9). During the experiment, it was really confirmed that the buoys could operate even at extremely high temperatures of the installation surface. This made it possible to prove that the developed equipment really has unique characteristics and capabilities, which makes it possible to use it both in conditions of extremely low and extremely high ambient temperatures.

**Figure 10:**
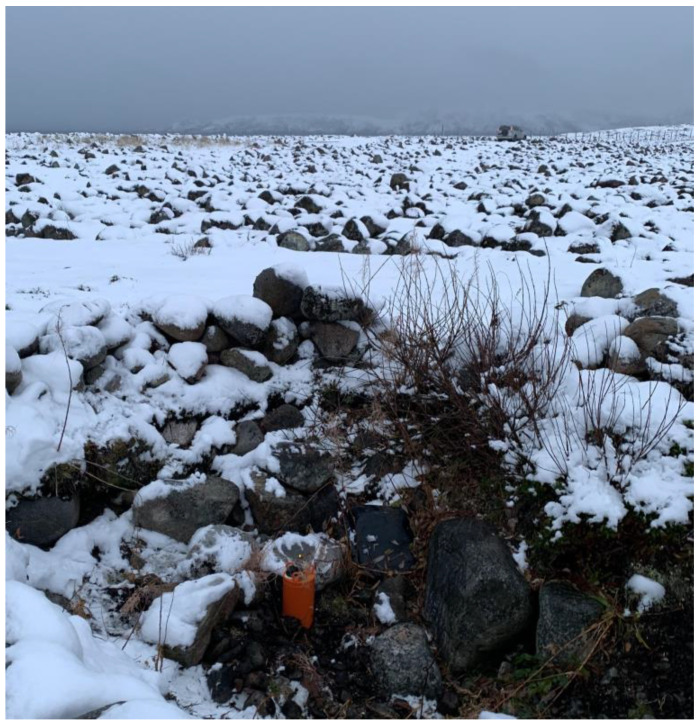
Installation of a geohydroacoustic buoy near the Teriberka village (Murmansk region, Russia) in frames of the Arctic expedition organized by the Russian Geographical Society in 2020.

Furthermore, during summer-autumn 2020, the presented buoys have found wide application within the framework of scientific research geophysical work carried out in the complex expedition of the Northern Navy of the Russian Federation and the Russian Geographical Society in the Arctic archipelagos along the Northern Sea Route (see Figure 10). Seismological work was carried out at 20 sites in the eastern and western Taimyr, the Nordenskjold archipelagos and the small islands of Shkher Minin, as well as on the Wrangel and Kotelny islands. Since the work of the expedition was just finished, it is not possible to present any received and processed data of field research within the framework of this article. 

The main thing that we wanted to especially note here is that the developed buoys were not only tested under ideal conditions of stationary seismic stations and laboratories, but also took part in the acquisition of geophysical data within the framework of several scientific expeditions, where its distinguished themselves by the reliability of work and the quality of the data obtained. 

More details about the results of the author’s research using these buoys can be found in the publications [30,31].

## 4. Discussion

A series of experimental works using the developed ice-class buoys, carried out in real arctic conditions, showed the versatility of the developed device, sufficient sensitivity, and dynamic range. Most of the experimental results have demonstrated that the operational frequency range of the broadband molecular–electronic velocimeter of the ice-based buoy is 0.03–50 Hz. At the same time, the key quality parameters of the hardware and software of the data acquisition system as well as the communication interfaces are quite reliable and the prototype well meets the high industry standards for modern seismology. It should be emphasized that innovative series of molecular–electronic sensors are suitable for a whole range of seismic surveys—land-based [29] and underwater [32], while original applications in ice conditions are in progress [33]. The ability to install various sensors in geohydroacoustic buoys allows multivariate monitoring of geohydroacoustic signals in a broadband frequency range. The presence of a temperature sensor makes it possible to take into account the influence of temperature on the measurement results. Another significant advantage of the ice buoy is the possibility of its long-term operation in autonomous mode. In all the studies carried out, the instruments were distinguished by their high reliability, consisting in complete reliability and quick operational entry into the normal mode, which was especially important during expedition works under difficult conditions of time constraints, requiring high speed and mobility during geophysical research.

The performance of the geohydroacoustic buoys has been tested in numerous laboratory and field conditions. This is evidenced by a comparison of the seismic record of our system with the data of such instruments as Streckeisen (STS-1V/VBB) and Güralp (CMG-3ESP). In the field, all these sensors were tested together when a seismic profile passed through the Kaluga ring structure [29] in accordance with the microseismic sounding technique [34]. At each observation point, the measurements were duplicated by very reliable SM3-OC instruments [35], widely used in Russia. A comparative analysis of the reconstructing results of the area deep structure showed that the performance of molecular–electronic sensors is not inferior to famous pendulum devices with a long history. We also compared the temperature dependence of the buoys with the previously mentioned SM3-OC. This comparison showed that the parameters of molecular-electronic seismometers are slightly dependent on temperature.

It should also be noted that buoys have a number of significant advantages associated primarily with the use of a molecular electronic sensing element. First of all, these devices have high operating qualities necessary for performing field work associated with frequent changes in the sensor installation site. This is facilitated by the absence of the need for locking, which significantly speeds up the installation set up procedure. Due to this feature, the buoy can be used for underwater measurements, and the simplicity of its design makes it much more accessible than complex bottom systems with hinged mechanisms and locking systems. The absence of precision mechanics elements increases the resistance of devices to shocks and other mechanical influences. The fact that buoys are weakly sensitive to the angle of inclination during installation played an important role, which is useful when installing the device on unstable ground, for example, the seabed or ice cover. The device is extremely easy to use—after setting the operating parameters, you just need to turn it on. Furthermore, moving the buoy to another measuring point (which is often required when carrying out field work) does not even require turning off the device’s power. Low power consumption allows to make measurements for more than a week without recharging the batteries. The connection of all modules (sensing molecular–electronic element, data acquisition system, battery, WiFi and GPS antenna) in a single case simplifies the work and increases reliability, since there are no extra wires and, as a result, connectors. 

The case, made of thick duralumin, provides high impact resistance and the ability to install the device in exceptional conditions, for example, on the ocean floor or ice. However, on the other hand, the case of the buoy gives it a lot of weight, which makes it difficult to carry without the use of transport. As a rule, one researcher is unlikely to be able to carry more than two buoys at the same time. Furthermore, the need to install antennas outside the metal case leads to a weakening of the point of its attachment; therefore, to use the buoy at great depths, you have to dismantle the antennas and use special plugs. This procedure is inconvenient and time consuming. The disadvantages of the buoy include the instability of the phase-amplitude characteristics of the sensitive element, which requires regular calibration. In addition, for a number of geophysical tasks, the operating frequency range of the sensitive element may be insufficient, since now it is 0.033–50 Hz.

## 5. Conclusions

This article describes a new geophysical buoy developed by us, which is a completely autonomous system for recording seismohydroacoustic data based on molecular electronic converters with the ability to connect other sensitive elements. Laboratory tests, as well as field tests, have shown that the measurement accuracy of these devices is not inferior to the leading modern models. A comparative analysis of the recorded time series featuring the microseismic noise and some distant earthquakes has confirmed that the buoy prototype meets the high standards of modern seismology. At the same time, the advantages of these buoys are much higher. Distinctive features of the developed geohydroacoustic buoys are

- Built-in 24-bit precise four channels data acquisition system;
- Quick start of measurement (data collection);
- Possibility to connect different types of sensitive elements;
- Ability to take into account ambient temperature and pressure;
- Low power consumption, which ensures autonomous operation of the buoy for at least one week;
- Possibility of installation on ice and under water at depths of up to 300 m;
- Low sensitivity to the angle of inclination during installation;
- Resistance to shock and mechanical stress;
- Absence of necessity to use special devices during transportation.

A significant advantage of the developed buoys is their rock-solid casing, so it possible to transport them without special handling, which is especially important during transportation by such special means as boats, helicopters, snowmobiles, etc. However, the other side of this advantage is the weight of the buoy leading to certain limitations in field works, as it was confirmed within the framework of the Arctic expedition organized recently by the Russian Geographical Society. In this regard, it seems convenient to develop a lightweight version of the buoy with fiberglass housing.

Summing up, we can conclude that this device is a highly effective tool for conducting field seismic observations for a wide range of tasks, such as the placement of temporary seismic observation points, including as part of microgroups, or conducting seismic surveys. The use of buoys in long-term seismic observatories is complicated by the need for annual calibration. Thus, the use of innovative technologies has made it possible to create fundamentally new approaches to the creation of a new compact seismic device capable to acquire seismohydroacoustic data in various environments, including icy seas in High Arctic.

## 6. Patents

Agafonov, V.M.; Avdyukhina, S.Y.; Egorov, E.V.; Sobisevich, A.L.; Sobisevich, L.E. Moscow Institute of Physics and Technology. Low frequency vector acoustic receiver. RF Patent RU2650839C1, 17 April 2018.

Dmitrichenko, V.P.; Presnov, D.A.; Rudenko, O.V.; Sobisevich, A.L.; Sobisevich, L.E. Sukhoparov, P.D.; Tikhotskii, S.A.; Shurup, A.S. Schmidt Institute of Physics of the Earth, Russian Academy of Sciences. Method of searching for mineral resources on the shelf of seas coated by ice. RF Patent RU2594663C1, 05 March 2018.

Sukhoparov, P.D.; Dmitrichenko, V.P.; Presnov, D.A.; Sobisevich, A.L.; Sobisevich, L.E. Schmidt Institute of Physics of the Earth, Russian Academy of Sciences. Three-component velocimeter. RF Patent RU2594663C1, 20 August 2016.

## Figures and Tables

**Table 1:** Basic parameters of the geohydroacoustic buoy.

Sensors Working Frequency Band: Hydrophones Vector Hydroacoustic Receiver Three Component Seismometer	1–2500 Hz 0.01–70 Hz 0.033–50 Hz
Integrated Weather Station: Monitoring	Internal temperature Atmospheric pressure
Integrated Data Acquisition System: Digital Capacity Effective Bits Sample Rate Built-in Memory Time Synchronization	24 bits 22 bits 1, 10, 50, 100, 250, 500, 1000 Hz 32 Gb GPS/GLONASS
Built-in Battery: Type Capacity Output Voltage	Li-ion 7000 mAh 12 V
External Connection	USB or WiFi
Overall Power Consumption	<500 mW
Duration of Autonomous Functioning	At least 168 h
Operational Temperature Range Normal Extended Version	−15 to +55 ℃ −40 to +55 ℃
Permissible Angle of Installation	±15°
Maximum Dipping Depth in Water	300 m
Ingress Protection Rating	IP67
Overall Dimension Diameter Height and Width	Ø 160 mm H × W: 450 mm × 130 mm
Weight	7 kg

**Table 2:** Parameters of molecular–electronic seismic sensor CME-4211V.

Frequency Band	0.033–50 Hz (T = 30-0.02 s)
Conversion Coefficient	2000 V/(m/s)
Maximum Recorded Signal	±5.0 mm/s
Integral Noise in 0.033–50 Hz Band	76 nm/s
Seismometer Power/Consumption	10.5–30 V DC/<12 mA (126 mW)
Operational Temperature Range	−40 to +55 ℃
Permissible Angle of Installation	±15°
Weight	0.4 kg

## References

[1] Rogozhin E.A., Antonovskaya G., Kapustian N.K. (2016). Current state and prospects of the development of an Arctic seismic monitoring system. Seism. Instrum..

[2] Asming V.E., Baranov S.V., Vinogradov Y.A., Voronin A.I. (2013). Seismic and infrasonic monitoring on the Spits-bergen archipelago. Seism. Instrum..

[3] Asming V.E., Baranov S., Vinogradov A.N., Vinogradov Y.A., Fedorov A.V. (2016). Using an infrasonic method to monitor the destruction of glaciers in Arctic conditions. Acoust. Phys..

[4] Vinogradov Y.A., Asming V.E., Baranov S., Fedorov A.V., Vinogradov A.N. (2015). Seismic and infrasonic monitoring of glacier destruction: A pilot experiment on Svalbard. Seism. Instrum..

[5] Walter F., Roux P., Roeoesli C., Lecointre A., Kilb D., Roux P.-F. (2015). Using glacier seismicity for phase velocity measurements and Green’s function retrieval. Geophys. J. Int..

[6] Gibbons S.J., Asming V., Eliasson L., Fedorov A., Fyen J., Kero J., Kozlovskaya E., Kvaerna T., Liszka L., Näsholm S.P. (2015). The European Arctic: A Laboratory for Seismoacoustic Studies. Seism. Res. Lett..

[7] Sobisevich A.L., Presnov D.A., Zhostkov R.A., Sobisevich L.E., Shurup A.S., Likhodeev D.V., Agafonov V.M. (2018). Geohydroacoustic noise monitoring of the under-ice water areas of the northern seas. Seism. Instrum..

[8] Gordienko V.A., Goncharenko B.I., Ilyushin Y. (1993). A Peculiarities of formation of the vector-phase structure of oceanic noise fields. Akust. Zh..

[9] D’Alessandro A., Scudero S., Vitale G. (2019). A Review of the Capacitive MEMS for Seismology. Sensors.

[10] Zheng X., Chen D., Wang J., Chen J., Xu C., Qi W., Liu B. (2019). Microelectromechanical System-Based Electrochemical Seismometers with Two Pairs of Electrodes Integrated on One Chip. Sensors.

[11] Kharlamov A. (2004). A Non-Traditional High Performance Seismic Sensor.

[12] Electrochemical Sensor Transducers. http://www.eentec.com/pdf/ELECTROCHEMICA-1.pdf/.

[13] Agafonov V.M., Egorov I.V., Shabalina A.S. (2014). Operating principles and technical characteristics of a small-sized molecular-electronic seismic sensor with negative feedback. Seism. Instrum..

[14] Agafonov V.M., Neeshpapa A.V., Shabalina A.S., Beer M., Kougioumtzoglou I.A., Patelli E., Siu-Kui Au I. (2015). Electrochemical seismometers of linear and angular motion. Encyclopedia of Earthquake Engineering.

[15] Kapustian N.K., Antonovskaya G., Agafonov V.M., Neumoin K., Safonov M. (2012). Seismic Monitoring of Linear and Rotational Oscillations of the Multistory Buildings in Moscow. Preliminary Reconnaissance Report of the 2011 Tohoku-Chiho Taiheiyo-Oki Earthquake.

[16] Kislov K.V., Gravirov V.V. Sensitivity of broadband seismic instrument parameters to environment. Proceedings of the 9th International Conference Problems of Geocosmos.

[17] Kislov K.V., Gravirov V.V. (2013). Investigation of the influence of the environment on the noise of broadband seismic equipment. Comp. Seismol..

[18] Sze S.M., Kwok K.N. (2006). Physics of Semiconductor Devices.

[19] Nyquist H. (1928). Thermal Agitation of Electric Charge in Conductors. Phys. Rev..

[20] Horowitz P., Winfield H. (1989). The Art of Electronics.

[21] Bryant J. (1990). Ask the Applications Engineer. Analog Dialogue.

[22] Van der Aldert Z., Hoffman J.G. (1960). Fluctuation Phenomena in Semi-Conductors. Phys. Today.

[23] Bonani F., Ghione G. (2001). Noise in Semiconductor Devices. 3D Microelectronic Packaging.

[24] Sutcliffe H. (1984). Noise in Electronic Devices and Systems. Electron. Power.

[25] Voss R.F. (1978). Linearity of 1f Noise Mechanisms. Phys. Rev. Lett..

[26] Wielandt E., Bormann P. (2012). Seismic Sensors and Their Calibration. New Manual of Seismological Observatory Practice (NMSOP-2).

[27] Streckeisen STS-2 Broadband Sensor. https://www.passcal.nmt.edu/content/instrumentation/sensors/broadband-sensors/sts-2-bb-sensor.

[28] Mitronovas W. (1976). Temperature effects on long-period seismographs: An accurate method to determine the transfer function. BSSA.

[29] Gorbenko V.I., Zhostkov R.A., Likhodeev D.V., Presnov D.A., Sobisevich A.L. (2017). Feasibility of using molecular-electronic seismometers in passive seismic prospecting: Deep structure of the Kaluga ring structure from microseismic sounding. Seism. Instrum..

[30] Konkov A.I., Manakov S.A., Presnov D.A. (2020). On the Coherence of Impulse Seismoacoustic Sources in Ice Conditions. Seism. Instrum..

[31] Presnov D.A., Sobisevich A.L., Shurup A.S. (2020). Research of Shallow Sea Passive Tomography Based on Ice Measurements Data. Bull. Russ. Acad. Sci. Phys..

[32] Antonov A.N., Avdyukhina S.Y., Egorov I.V., Zhostkov R.A., Likhodeev D.V., Presnov D.A., Shabalina A.S. A Broadband Seismic Station for Seismic Surveys on the sea Bottom and in Transitional Zone, Designed on the Basis Molecular Electron Sensors. Proceedings of the Research and Practice Conference Seismic Technologies.

[33] Presnov D.A., Zhostkov R.A., Shurup A.S., Sobisevich A.L. (2017). On-site observations of seismoacoustic waves under the conditions of an ice-covered water medium. Bull. Russ. Acad. Sci. Phys..

[34] Gorbatikov A.V. (2006). Method of Seismic Exploration. Patent.

[35] Sarkgsyan R.E. (2014). Modernization of the SM-3 seismometer. Seism. Instrum..

